# Nik-related kinase regulates trophoblast proliferation and placental development by modulating AKT phosphorylation

**DOI:** 10.1371/journal.pone.0171503

**Published:** 2017-02-02

**Authors:** Yuka Morioka, Jin-Min Nam, Takashi Ohashi

**Affiliations:** 1 Division of Disease Model Innovation, Institute for Genetic Medicine, Hokkaido University, Sapporo, Hokkaido, Japan; 2 Global Station for Quantum Medical Science and Engineering, Global Institution for Collaborative Research and Education (GI-CoRE), Hokkaido University, Sapporo, Hokkaido, Japan; Academic Medical Centre, University of Amsterdam, NETHERLANDS

## Abstract

Nik-related kinase (Nrk) is a Ser/Thr kinase and was initially discovered as a molecule that was predominantly detected in skeletal muscles during development. A recent study using *Nrk*-null mice suggested the importance of *Nrk* in proper placental development; however, the molecular mechanism remains unknown. In this study, we demonstrated that differentiated trophoblasts from murine embryonic stem cells (ESCs) endogenously expressed *Nrk* and that *Nrk* disruption led to the enhanced proliferation of differentiated trophoblasts. This phenomenon may reflect the overproliferation of trophoblasts that has been reported in enlarged placentas of *Nrk*-null mice. Furthermore, we demonstrated that AKT phosphorylation at Ser473 was upregulated in *Nrk*-null trophoblasts and that inhibition of AKT phosphorylation cancelled the enhanced proliferation observed in differentiated *Nrk*-null trophoblasts. These results indicated that the upregulation of AKT phosphorylation was the possible cause of enhanced proliferation observed in *Nrk*-null trophoblasts. The upregulation of AKT phosphorylation was also confirmed in enlarged *Nrk*-null placentas *in vivo*, suggesting that proper regulation of AKT by *Nrk* was important for normal placental development. In addition, our detailed analysis on phosphorylation status of AKT isoforms in newly established trophoblast stem cells (TSCs) revealed that different levels of upregulation of AKT phosphorylation were occurred in *Nrk*-null TSCs depending on AKT isoforms. These results further support the importance of *Nrk* in proper development of trophoblast lineage cells and indicate the possible application of TSCs for the analysis of differently regulated activation mechanisms of AKT isoforms.

## Introduction

The placenta is a unique and complicated organ that plays an essential role in supporting foetal development through its involvement in various functions such as gas exchange, nutrient supply and hormone production during gestation. Because placental defects are associated with many pregnancy-related disorders [1, 2], a better understanding of placental development is important to overcome these clinical problems. Animal models have been used to elucidate the underlying mechanisms of human disorders. From the 1990s to date, numerous gene knockout mice have been generated. Among them, >100 mutant mice have placental defects and have substantially contributed to the advancing of our knowledge regarding the genetic control of placental development [1, 3]. However, further investigations are necessary for the complete understanding of placental development.

Placentomegaly is a major placental abnormality that is associated with several foetal disorders and is present in various gene-manipulated mice, including those with *Igf2*, *H19*, *Cdkn1* and *Phlda2* malfunctions [4–6]. These genes are well conserved in humans and are included in a large cluster of imprinted genes that are located on mouse chromosome 7. Dysregulation of this imprinted region may be associated with Beckwith–Widemann syndrome, which is also known as an overgrowth syndrome that exhibits placentomegaly [5–8]. In addition, previous studies suggested important roles of X-linked genes, such as *Gpc3* [9], *Esx1* [10], *Plac1* [11] and *Nrk* [12], in regulating normal placental development because mice that lacked any of these X-linked genes had enlarged placentas [9–12]. The human *GPC3* gene is responsible for the type 1 Simpson–Golabi–Behmel syndrome, which is another overgrowth syndrome that is characterised by placentomegaly [9]. Moreover, cloned mice that were generated by somatic cell nuclear transfer exhibited placentomegaly with a reduced expression of a number of X-linked genes [13, 14]. Because these previous reports revealed that many placentomegaly-related genes were present on either chromosome 7 or the X chromosome in mice, further studies of the genes on these chromosomes are important to understand the pathogenesis of pregnancy-related disorders.

One of the placentomegaly-related X-linked genes, *Nik-related kinase* (*Nrk*), encodes a Ser/Thr kinase that belongs to the germinal centre kinase family. *Nrk* was originally cloned as a gene that was expressed only in the skeletal muscle during development [15, 16]. However, Denda *et al*. [12] recently reported that *Nrk* was also expressed in the spongiotrophoblast layer of the murine placenta and that *NRK* expression was also detected in the human placenta. This study further demonstrated a high frequency of neonatal death, delivery defects and placental overgrowth in *Nrk-*null mice, suggesting a novel role of *Nrk* in reproduction.

Because placentomegaly has been closely related to pregnancy-related disorders, we aimed to elucidate the mechanism through which *Nrk* deficiency induced placental enlargement. Previous studies regarding overexpression experiments in cultured cells have suggested that *Nrk* participated in JNK signalling pathway activation through the MEKK1 and MKK4 kinase cascade [16], promotion of apoptosis through JNK activation [17] and polymerization of actin through cofilin phosphorylation [18]. Because these phenomena have been observed in cells that ectopically expressed *Nrk*, further studies using placental trophoblast cells that endogenously express *Nrk* are desirable to better understand the molecular functions of *Nrk* in placental development. The caudal-related homeobox protein CDX2 is the earliest trophoblast-specific transcription factor, and *Cdx2* overexpression can induce trophoblast lineages from pluripotent murine embryonic stem cells (ESCs) [19, 20]. Furthermore, Hayashi *et al*. [21] recently reported that ESCs were able to differentiate into trophoblast lineages on laminin-coated dishes in a BMP4-containing medium through *Cdx2* expression. Therefore, this culture system could be applicable for examining the role of *Nrk* in trophoblasts during placental development. In the present study, we further modified this culture system and demonstrated that *Nrk* deficiency resulted in enhanced trophoblast proliferation, which could reflect the enlarged placenta observed in *Nrk*-null mice. Furthermore, we found that enhanced proliferation in *Nrk*-null trophoblasts was associated with the upregulation of AKT phosphorylation at Ser473. We also established murine trophoblast stem cells (TSCs) culture system that is known as an excellent tool for deciphering gene function in trophoblasts [22] and explored the involvement of *Nrk* in AKT activation and trophoblast development. Our results indicated that NRK expression was responsible for the regulation of proper AKT activation in TSCs, which was similarly observed in ESC-derived trophoblasts. Moreover, the upregulation of AKT phosphorylation was confirmed in enlarged placentas that were observed in *Nrk*-null mice *in vivo*, suggesting an important role of *Nrk* in maintaining proper AKT activation during placental development.

## Methods

### Cell culture

EGR-G101 ESC line was derived from a male C57BL/6N mouse blastocyst that expressed a green fluorescent protein [23]. A feeder-dependent EGR-G101 was routinely cultured [23] and later adapted for a feeder-independent culture as previously reported [24]. Feeder-independent ESCs were maintained on type I collagen-coated (Nitta gelatin, Osaka, Japan) dishes in a defined ESF7 medium containing a supernatant of mLIF-producing cells. Inducing differentiation of feeder-independent ESCs to trophoblasts was performed as previously reported [21]. Briefly, feeder-independent ESCs were seeded on laminin-coated (Sigma-Aldrich, St. Louis, MO) dishes in a defined ESF5 medium containing rhBMP4 (BioLegend, San Diego, CA). After 4 days of culture, the cells reached confluency and were then subcultured for an additional 4 days before finally differentiating to trophoblasts.

Mouse TSC lines from wild-type and *Nrk*-null embryos were established from expanded blastocysts as described previously [22]. Undifferentiated TSCs were cultured in TSC medium [RPMI1640 (Nacalai Tesque, Kyoto, Japan) supplemented with 20% fetal bovine serum (Biowest, Riverside, MO), 1 mM sodium pyruvate (Nacalai Tesque), 0.1 mM β-mercaptoethanol (Thermo Fisher Scientific, Waltham, MA), 2 mM L-glutamine (Nacalai Tesque) and 100 U/ml penicillin and 0.1 mg/ml streptomycin (Nacalai Tesque)] containing 70% mouse embryonic fibroblast-conditioned medium (MEF-CM), 25 ng/ml hFGF4 (BioLegend) and 1 μg ml/ml heparin (Sigma-Aldrich). Differentiation of TSCs was induced by culturing the cells in TSC medium without MEF-CM, hFGF4 and heparin for 6 days.

### RNA isolation and RT-PCR analysis

Total RNA was extracted using TRIzol (Thermo Fisher Scientific) from three independent cultures of each cell type, then 0.5 μg of each RNA sample was reverse transcribed using the ReverTraAce (Toyobo, Osaka, Japan). One twentieth of each cDNA sample was subjected to PCR analysis. Conventional PCR followed by gel electrophoresis was performed using the KOD FX (Toyobo) on the Thermal Cycler Dice Touch (Takara bio, Shiga, Japan). Real-time PCR was performed using the THUNDERBIRD SYBR qPCR Mix (Toyobo) on the StepOne Real-Time PCR System (Thermo Fisher Scientific). Data were analyzed by Comparative CT Method using StepOne software and the relative expression level of each target gene mRNA was normalized to the amount of *Gapdh* control. Each reaction was performed in triplicate and data are presented as the mean plus SD. The primer sets used in this study are shown in Table 1.

**Table 1:** List of primer sequences.

Name	Sequence
Nrk-F	5′-tagtggattttggagtgagtgc-3′
Nrk-R	5′-cttctgtaatcataggaacacc-3′
Plf-F	5′-aggaacaagccaggctcaca-3′
Plf-R	5′-ttccggactgcgttgatctt-3′
Tpbpa-F	5′-acagccagttgttgatgacc-3′
Tpbpa-R	5′-caggatcccacttgtcagg-3′
Hand1-F	5′-cgtgagtgcatccccaatg-3′
Hand1-R	5′-gccagcacgtccatcaagtag-3′
Psx1-F	5′-cgatggatgggtgtggatga-3′
Psx1-R	5′-tgacagggctggcactcaag-3′
Mash2-F	5′-cgggatctgcactcgaggat-3′
Mash2-R	5′-ggtgggaagtggacgtttgc-3′
Esx1-F	5′-gagctggaggcctttttcca-3′
Esx1-R	5′-acacccacagggggactcat-3′
Ets2-F	5′-ctcggctcaacaccgtcaat-3′
Ets2-R	5′-agctgtccccaccgttctct-3′
Pl2-F	5′-ctgctcttccacatgtacct-3′
Pl2-R	5′-taagagcactgttcttagtgg-3′
Essrb-F	5′-atggatgaggaacactctcg-3′
Essrb-R	5′-cagctcatagtcctgcagc-3′
Gapdh-F	5′-tgtgtccgtcgtggatctga-3′
Gapdh-R	5′-ttgctgttgaagtcgcaggag-3′
5′-F	5′-actggtgaatttggagcagtcaaaacttcc-3′
5′-R	5′-cttccccacaacgggttcttctgttagtcc-3′
3′-F	5′-ggttgatatctctatagtcgcagtaggcgg-3′
3′-R	5′-ttgtggaccggacaagtaatcctgcagtcg-3′
F	5′-acaccaagtaattcaccgggacatcaaagg-3′
WT-R	5′-gtcttagatagtctttcagaaagcatcg-3′
KO-R	5′-cttccccacaacgggttcttctgttagtcc-3′

### Generation of *Nrk*-null ESC

The targeting vector PRPGS00061_B_H04 was purchased from the International Knock-Out Mouse Program. Gene targeting in feeder-dependent EGR-G101 ESCs was performed as previously described [25, 26]. Briefly, single cell suspensions (0.8 mL of 10^7^ cells/mL) were mixed with 20 μg of AsiSI-digested linear-targeting vector and were subjected to electroporation at a pulse of 250 V and 500 μF using a Gene Pulser II (Bio-Rad, Hercules, CA). The mutated cells were selected with G418 (150 μg/mL), and the homologous recombination events were detected using PCR amplification with the following primers: 5′-F and 5′-R for the 5′-arm and 3′-F and 3′-R for the 3′-arm. The primer sequences are shown in Table 1.

### Cell proliferation assay

Cell proliferation was evaluated by counting the number of cells. Cells were washed with PBS and were then dissociated with TrypLE^TM^ Express Enzyme (Thermo Fisher Scientific). The dissociated cell suspensions were mixed with an equal volume of 0.5% trypan blue stain solution (Nacalai Tesque), and the cell numbers were counted using the Luna^TM^ automated cell counter (Logos Biosystems, Annandale, VA). The proliferation rate was calculated relative to the cell numbers of the day 1 controls. The value of day 1 was indicated as 1.

### Western blotting

To prepare whole-cell extracts, cells were first washed with ice-cold PBS and were then lysed in RIPA buffer [1% Nonidet P-40, 150 mM NaCl, 50 mM Tris-HCl (pH7.5), 1 mM EDTA and 0.5% sodium deoxycholate] that contained the cOmplete^TM^, Mini protease inhibitor cocktail (Roche, Basel, Switzerland) and the PhosSTOP^TM^ phosphatase inhibitor cocktail (Roche) on ice. After being passed through a 26-gauge needle 10 times, cell extracts were centrifuged and the supernatants were collected. To prepare the extracts of E18.5 mouse placentas, which had been stored at −80°C, the tissues were disrupted using a bead crusher μT-01 (TAITEC, Saitama, Japan) for 15 s at maximum speeds in ice-cold RIPA buffer that was supplemented with protease and phosphatase inhibitors. After centrifugation, the supernatants were collected as placental extracts. The protein concentration of each sample was determined using the Quick Start^TM^ Bradford 1x Dye Reagent and the Quick Start^TM^ Bovine Serum Albumin Standard Set (Bio-Rad), according to the manufacturer’s instructions.

Whole-cell extracts (30 μg) or placental extracts (100 μg) were separated using SDS-PAGE with 12% gel and subsequently transferred onto a polyvinylidene fluoride membrane. The membranes were blocked with TBST buffer [25 mM Tris-HCl (pH 7.5), 137 mM NaCl, 2.5 mM KCl and 0.1% Tween-20] that contained 5% skim milk. After washing with TBST, the blocked membranes were incubated overnight at 4°C with a primary antibody in TBST that contained 5% BSA. After washing with TBST, the membranes were incubated for 1 h at room temperature with a secondary antibody in TBST that contained 5% skim milk. After washing with TBST, the protein–antibody complex was detected using the Chemi-Lumi One L (Nacalai Tesque) and the ImageQuant^TM^ LAS 4000 mini imaging system (GE Healthcare Japan, Tokyo, Japan), according to the manufacturer’s instructions. The signal density was analyzed by ImageQuant^TM^ TL software. As needed, membranes were stripped by incubation at 50°C in SDS-based stripping buffer [62.5 mM Tris-HCl (pH 6.8), 2% SDS and 100 mM β-mercapthoethanol] for 30 min to detect other proteins.

### Immunoprecipitation

For immunoprecipitation, 1 mg/ml whole-cell extracts were prepared as described above. The 250 μg proteins were incubated for 3 h at 4°C with the indicated antibody under constant rotation. Then Protein A-coated magnetic beads (Bio-Rad) were added and mixed samples were incubated overnight at 4°C under constant rotation. Magnetic beads were washed 3 times with ice-cold RIPA buffer supplemented with phosphatase inhibitors and the binding proteins were eluted using SDS-PAGE loading buffer for western blotting.

### Antibodies

Anti-NRK rabbit polyclonal primary antibodies were generated against a synthetic peptide (CGAAGYNGGDVGGNHGAAFN), and then purified with a column immobilized with antigen peptides (Sigma-Aldrich). We confirmed that the newly established polyclonal antibodies are able to detect several proteins in wild-type cells, but not in *Nrk*-null cells. Because it has been reported that ectopically expressed NRK in culture cells is cleaved by caspases [17], the largest protein probably corresponds to full-length NRK and others are judged to be the cleaved fragments of NRK proteins. The antibodies also recognize several non-specific proteins that are present in both wild-type and *N*rk-null cells. These anti-NRK antibodies were used at a 1:4,000 dilution for western blotting. The commercially available primary and secondary antibodies used in this study are summarized in Table 2.

**Table 2:** List of antibodies.

**Primary antibodies**			
**Name**	**Company**	**Catalog No.**	**Dilution**
Phospho-Akt (Ser473)	CST	#4060	1:2,000 (WB)1:50 (IH)
Phospho-Akt (Thr308)	CST	#13038	1:1,000 (WB)
Akt (pan)	CST	#4691	1:1,000 (WB)1:50 (IH)
Akt1	CST	#2938	1:1,000 (WB)1:50 (IP)
Akt2	CST	#3063	1:1,000 (WB)1:100 (IP)
Akt3	CST	#14982	1:1,000 (WB)1:100 (IP)
Phospho-Erk1/2 (Thr202/Tyr204)	CST	#4370	1:2,000 (WB)
Erk1/2	CST	#4695	1:1,000 (WB)
Phospho-SAPK/JNK (Thr183/Tyr185)	CST	#9251	1:1,000 (WB)
SAPK/JNK	CST	#9252	1:1,000 (WB)
β-Actin	Sigma-Aldrich	A5441	1:5,000 (WB)
**Secondary antibodies**			
**Name**	**Company**	**Catalog No.**	**Dilution**
HRP-conjugated goat anti-rabbit IgG	Jackson IR	111-035-003	1:5,000
HRP-conjugated donkey anti-mouse IgG	Jackson IR	715-036-151	1:20,000

CST: Cell Signaling Technology, Jackson IR: Jackson ImmunoResearch, WB: western blotting, IH: immunohistochemistry, IP: immunoprecipitation

### Inhibition of AKT phosphorylation

The AKT inhibitor MK-2206 (Cayman Chemical, Ann Arbor, MI) was dissolved in DMSO solution, stored at −80°C as a stock solution (5 mM) and then diluted with a culture medium prior to use. Undifferentiated ESCs were seeded on a laminin-coated 24-well plate at 10^6^ cells/well in an rhBMP4-supplemented ESF5 medium, and the adherent cell number was counted 1 day after seeding. The cells were treated at 0–500 nM of MK-2206 on day 2 and maintained in this culture condition until day 4 (n = 8). Four days after seeding, the cell number was counted (n = 4) and the proliferation rate was calculated relative to the cell numbers of the untreated day 1 controls. The value of day1 was indicated as 1. The remaining cells (n = 4) were collected together, and western blotting was performed to assess the AKT Ser473 phosphorylation status.

### Animals

Wild-type C57BL/6N and ICR mice were purchased from Japan SLC (Shizuoka, Japan) and were maintained under specific-pathogen free conditions. The mice were housed in plastic cages with wire mesh lids and water bottles (CLEA Japan, Tokyo, Japan), and were put on the open racks. Mice were fed Labo MR Stock diet (Nosan, Kanagawa, Japan) and maintained on wood chip bedding (Iwakura, Hokkaido, Japan). All animals were observed daily for signs of illness or abnormal behavior. All animal experiments were conducted in accordance with the Hokkaido University Guide for the Regulation on Animal Experimentation. All experiments were reviewed and approved by the Animal Care and Use Committee of the Hokkaido University (Permit Number: 10–0114 and 14–0143). All surgery was performed under sodium pentobarbital anesthesia, and all efforts were made to minimize suffering. Prior to our experimental endpoint, no mice became severely ill or died. In our experimental endpoint, all mice were euthanized by a high-dose (>120 mg/kg) intraperitoneal administration of sodium pentobarbital.

### Generation of *Nrk*-null mice

We generated chimeric mice by aggregation of *Nrk*-null ESCs and 8-cell embryos, followed by transplantation into pseudopregnant ICR females as previously described [27]. Briefly, 8-week-old wild-type ICR females were superovulated and then mated with wild-type ICR males. Two-cell stage embryos were collected from the females at 1.5 days after copulation and then incubated in KSOM-AA medium for 1 day to obtain 8-cell embryos. The zona pellucida of the 8-cell embryos was removed with acidic Tyrode’s solution (Sigma-Aldrich) [28]. These 8-cell embryos without zona pellucida were individually incubated in 5 μL of KSOM-AA microdrops that contained 5–10 ESCs for 1 day to obtain blastocysts. We transplanted 10 blastocysts into each horn of the uterus of pseudopregnant ICR females. The chimeric male mice were mated with wild-type C57BL/6N females, and the germ line transmission was confirmed by observing green fluorescence in the neonates [29]. Mice were genotyped using PCR amplification with the following primers: F and WT-R for detection of the wild-type allele. F and KO-R for detection of the targeted allele. The primer sequences are shown in Table 1.

### Histological and immunohistochemical analysis

E18.5 placentas were fixed in 4% paraformaldehyde in PBS and were processed for paraffin embedding. For histological analysis, the 4-μm paraffin sections were stained with periodic acid-Schiff (PAS) and then counterstained with hematoxylin. For immunohistochemical analysis, the 4-μm paraffin sections were incubated overnight at 4°C with indicated primary antibodies. After incubation with secondary antibodies conjugated with horseradish peroxidase, sections were stained with diaminobenzidine tetrahydrochloride and then counterstained with hematoxylin.

### Statistics

Data were presented as mean plus SD. Groups were compared using the two-tailed Student’s *t*-test. We considered *P* values of <0.01 to be significant.

## Results

### Establishment of an *in vitro* culture system for investigating the role of *Nrk* in trophoblast proliferation and differentiation

**Fig 1:**
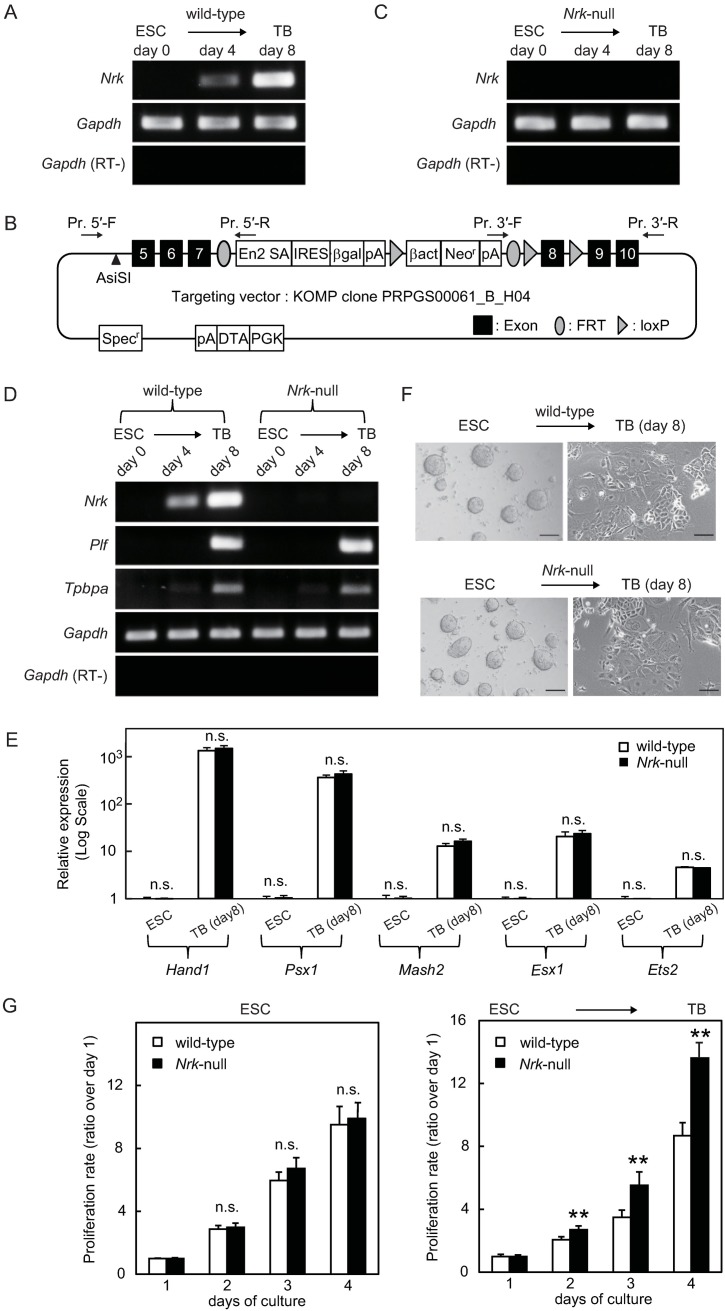
Disruption of *Nrk* resulted in enhanced proliferation of trophoblasts without obvious influence on their differentiation. (A) *Nrk* expression in wild-type cells was examined using RT-PCR analysis. Total RNAs were isolated from undifferentiated ESCs (day 0) and differentiated trophoblasts (day 4 and day 8) and were subjected to RT-PCR amplification. As an internal control, *Gapdh* was also amplified. RT-PCR amplification of *Gapdh* was also performed in the absence of reverse transcriptase (RT-). (B) Construct of the gene-targeting vector for *Nrk* disruption. The endogenous *Nrk* expression was disturbed by a gene-trapping cassette inserted between exon 7 and 8 of *Nrk* gene. The arrowhead indicates AsiSI recognition site for linearization of the vector. The arrows indicate PCR primers for detection of the homologous recombination events. (C) Deficiency of *Nrk* expression in *Nrk*-null cells was confirmed using RT-PCR analysis. Total RNAs were isolated from *Nrk*-null cells and were subsequently subjected to RT-PCR amplification as described in (A). (D) RT-PCR analysis of trophoblast marker gene expressions in wild-type and *Nrk*-null cells. Total RNAs were isolated from undifferentiated ESCs (day 0) and differentiated trophoblasts (day 4 and day 8) and were subsequently subjected to RT-PCR amplification as described in (A). (E) Real-time PCR analysis of transcription factor expressions in wild-type and *Nrk*-null cells. Undifferentiated ESCs and differentiated trophoblasts (day 8) were analyzed (n = 3). The examined gene expressions were normalized to the amount of *Gapdh* control. Each gene expression in undifferentiated wild-type ESCs is indicated as 1. Data are presented as mean plus SD. n.s. = no significant difference. (F) Phase-contrast photomicrographs of undifferentiated ESCs (left) and differentiated trophoblasts at day 8 (right) with or without *Nrk*. Scale bars indicate 200 μm. (G) Proliferation of undifferentiated ESCs and differentiated ESCs into trophoblasts. Undifferentiated ESCs were seeded on a type I collagen-coated 24-well plate at 10^6^ cells/well in a LIF-supplemented ESF7 medium (left) in which undifferentiated state of ESCs was maintained. Undifferentiated ESCs were seeded on a laminin-coated 24-well plate at 10^6^ cells/well in an rhBMP4-supplemented ESF5 medium (right) in which ESCs were induced to differentiate into trophoblasts. The cells were counted every 24 h (n = 6). The value of day1 is indicated as 1. Data are presented as mean plus SD. **P<0.01 compared to wild-type. n.s. = no significant difference. Shown are cropped gels. The gels with indicated cropping lines are shown in S1 Fig. ESC: embryonic stem cell, TB: trophoblast.

To investigate the precise roles of *Nrk* in trophoblast proliferation and differentiation, we first attempted to establish an *in vitro* culture system, wherein we could observe *Nrk* expression during trophoblast differentiation. As shown in Fig 1A, *Nrk* mRNA expression was not detected in undifferentiated ESCs. After initiating trophoblast differentiation, *Nrk* mRNA expression was faintly detected on the fourth day of culture and was clearly induced after 8 days of culture. These results indicated that this culture system could be applicable for examining the role of *Nrk* during ESC differentiation to trophoblasts.

To further understand this role, we generated *Nrk*-mutated EGR-G101 cells by homologous recombination with the gene-targeting vector shown in Fig 1B. We believed that a gene-trapping cassette that was inserted between exon 7 and 8 of *Nrk* gene would disrupt the endogenous *Nrk* expression. EGR-G101 cells with an *Nrk*-destructive mutation on the X chromosome were found to be incapable of producing NRK proteins because EGR-G101 cells have an XY genotype. To select *Nrk*-mutated cells, we assessed the homologous recombination using PCR analysis and then examined the ability of the mutated cells to express *Nrk* mRNA using RT-PCR analysis. In contrast to the clear induction of *Nrk* mRNA in wild-type EGR-G101 cells (Fig 1A), mutant EGR-G101 cells did not express *Nrk* mRNA even after 8 days of culture under differentiation-inducing conditions (Fig 1C), indicating *Nrk* deficiency in the mutant cells. Thus, we created an *in vitro* culture system wherein we could observe trophoblast proliferation and differentiation both in the presence and absence of *Nrk*.

### Disruption of *Nrk* did not show obvious influence on the differentiation from ESC to trophoblast

To examine whether ESC differentiation to trophoblasts was influenced by *Nrk* expression, we next compared trophoblast marker gene (*Plf* [30] and *Tpbpa* [31]) expressions, which were absent in ESCs, between wild-type and *Nrk*-null cells during trophoblast differentiation using RT-PCR analysis. As shown in Fig 1D, we found that the expression of both marker genes was similarly induced during trophoblast differentiation, regardless of the presence or absence of *Nrk*. In addition, we performed real-time PCR analysis to assess transcription factor (*Hand1* [32], *Psx1* [33], *Mash2* [34], *Esx1* [35] and *Ets2* [36]) expressions. These genes had been detected even in undifferentiated ESCs and had been enhanced during trophoblast differentiation. Our results demonstrated that the relative mRNA levels were increased approximately 4- to 1,500-fold in the differentiated trophoblasts on day 8 as compared with those in ESCs and that a similar degree of increase in each transcription factor was induced in both wild-type and *Nrk-*null cells during differentiation (Fig 1E). Moreover, we observed that the morphological changes during differentiation were not affected by *Nrk* deficiency (Fig 1F). These results suggested that *Nrk* did not play a critical role in the differentiation process from ESCs to trophoblasts.

### Disruption of *Nrk* resulted in enhanced proliferation of trophoblasts, which was associated with the upregulation of AKT Ser473 phosphorylation

Next, we examined whether *Nrk* deficiency affected cell proliferation. After having seeded ESCs, the cell numbers were counted every day until day 4 of culture, and the proliferation rate was calculated relative to the cell numbers on day 1. To avoid cell passaging during a proliferation assay, we thus decided to detect cell proliferation until day 4. Indeed, we confirmed *Nrk* expression on day 4 in wild-type cells (Fig 1A) and thus believed that a culture period of 4 days is enough to detect the difference of the cell proliferation between wild-type and *Nrk*-null cells. Our analysis of undifferentiated ESCs revealed equivalent levels of proliferation regardless of *Nrk* genotype (Fig 1G, left), which was consistent with the fact that *Nrk* was not detected in ESCs as shown in Fig 1A. In contrast, when we cultivated ESCs under differentiation-inducing conditions, a significant increase in proliferation was observed in *Nrk*-null cells on day 2 as compared with that in wild-type cells and was maintained until day 4 of culture (Fig 1G, right).

**Fig 2:**
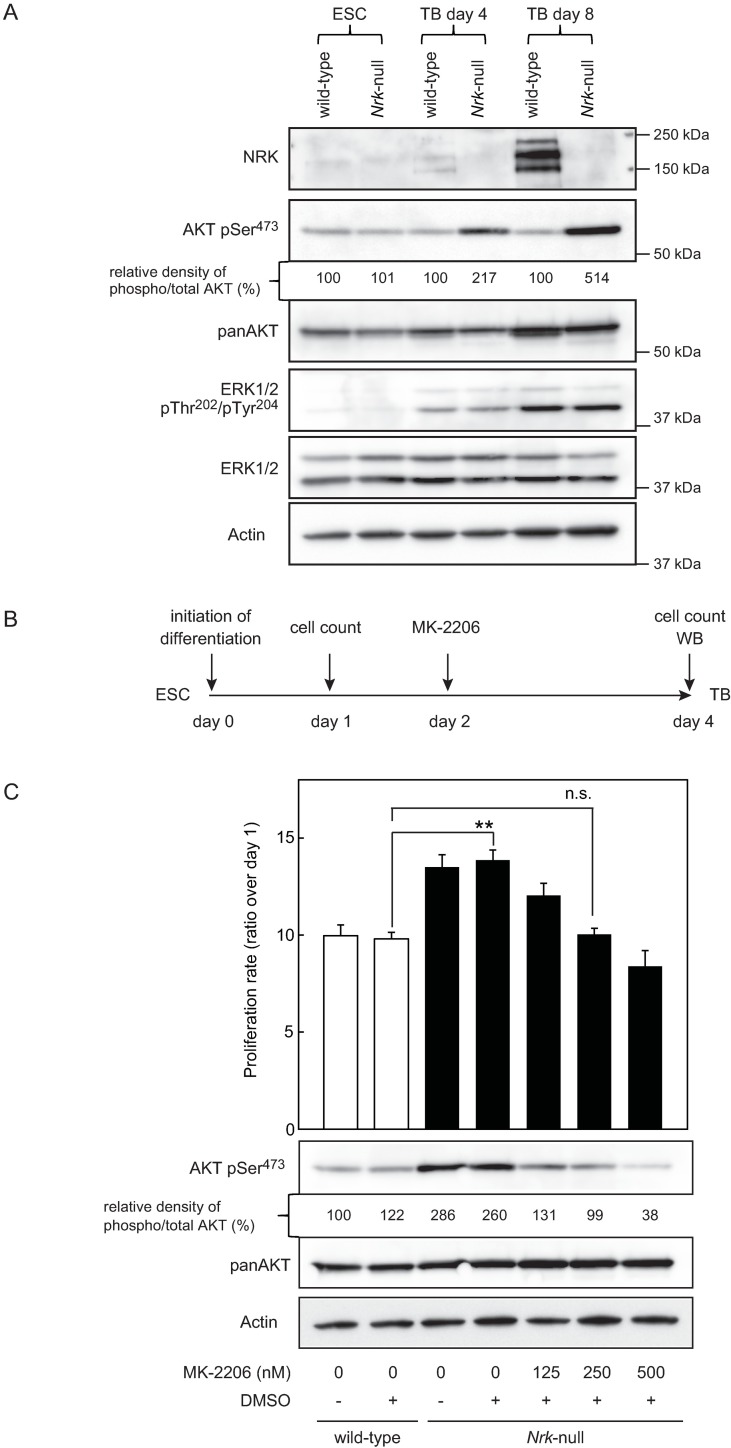
Enhanced proliferation observed in differentiated *Nrk*-null trophoblasts was associated with the upregulation of AKT Ser473 phosphorylation. (A) Western blot analysis of whole-cell extracts from ESCs and trophoblasts. Expressions of NRK, phosphorylated AKT at Ser473, total AKT, phosphorylated ERK1/2 at Thr202/Tyr204 and total ERK1/2 were compared between wild-type and *Nrk*-null cells. As an internal control, β-Actin was also detected. The relative density of phosphorylated vs total AKT was analyzed and was normalized to the value of wild-type. (B) The schematic representation of the experimental procedure to examine the effect of AKT inhibitor MK-2206. (C) Dose dependent effect of MK-2206 on the proliferation and AKT Ser473 phosphorylation in the differentiated *Nrk*-null trophoblasts. The proliferation rate of the trophoblasts on day 4 was calculated relative to the untreated day 1 control (n = 4) (top). Data are presented as mean plus SD. **P<0.01compared to wild-type. n.s. = no significant difference. Expression of phosphorylated AKT at Ser473 and total AKT in trophoblasts on day 4 was analyzed by western blotting (bottom). As an internal control, β-Actin was also detected. The relative density of phosphorylated vs total AKT was analyzed and was normalized to the value of untreated wild-type control. Shown are cropped blots. The blots with indicated cropping lines are shown in S3 Fig. WB: western blotting, ESC: embryonic stem cell, TB: trophoblast.

To explore the molecular mechanism of enhanced proliferation that was observed in *Nrk*-null trophoblasts, we first examined the phosphorylation status of JNK using western blotting. Although previous studies have suggested that *Nrk* participated in JNK activation [16–17], we detected no significant difference in JNK phosphorylation between wild-type and *Nrk*-null cells as shown in S2 Fig. This was consistent with a previous report by Denda *et al*. describing that the levels of JNK phosphorylation in placentas were equivalent between wild-type and *Nrk*-null embryos [12]. Next, we examined the phosphorylation statuses of AKT and ERK1/2 that have both been associated with placental development [37, 38] using western blotting. As shown in Fig 2A, we confirmed the induction of NRK protein expression in differentiated trophoblasts that were derived from wild-type ESCs but not from *Nrk*-null ESCs; this was consistent with the mRNA expression patterns shown in Fig 1D. Under these conditions, we found that AKT phosphorylation at Ser473 was upregulated in *Nrk*-null trophoblasts as compared with that in the wild-type controls. This was not due to the enhanced expression of total AKT proteins because we assessed the total AKT protein levels and failed to detect any significant differences between wild-type and *Nrk*-null trophoblasts. An anti-panAKT monoclonal antibody used in this study recognises all three AKT isoforms (AKT1, AKT2 and AKT3). Calculation of relative densities of phosphorylated vs total AKT revealed 2- to 5-fold increment of AKT Ser473 phosphorylation in *Nrk*-null trophoblasts as compared with that in wild-type control. We further assessed ERK1/2 phosphorylation and found no significant differences between wild-type and *Nrk*-null cells during the experiment, although a similar induction of ERK1/2 phosphorylation was observed in both types of cells during trophoblast differentiation. Thus, ERK1/2 phosphorylation may also be associated with trophoblast differentiation but may not be involved in the enhanced trophoblast proliferation that was observed in *Nrk*-null cells. Altogether, our results strongly suggested that enhanced proliferation observed in *Nrk*-null trophoblasts was caused by the upregulation of AKT Ser473 phosphorylation.

### Inhibition of AKT Ser473 phosphorylation cancelled the enhanced proliferation observed in differentiated *Nrk*-null cells

To further confirm whether excessive and non-physiological AKT Ser473 phosphorylation was responsible for enhanced proliferation of *Nrk*-null trophoblasts, we assessed the effect of an AKT-specific allosteric inhibitor, MK-2206, on *Nrk*-null trophoblast proliferation. MK-2206 binds to AKT, resulting in a decrease in AKT phosphorylation in a non-ATP competitive manner [39]. The schematic representation of the experimental procedure is shown in Fig 2B. The effects of MK-2206 were evaluated on day 4 by cell counting and western blotting. When we added various doses of MK-2206 dissolved in DMSO to the culture of differentiating *Nrk*-null trophoblasts on day 2, a dose-dependent proliferation inhibition was clearly observed on day 4 (Fig 2C, top). It was of note that this decrease in cell proliferation was accompanied by a reduction in AKT Ser473 phosphorylation. In particular, it appeared that 250 nM of MK-2206 was enough to reduce the level of AKT Ser473 phosphorylation in the *Nrk*-null cells to the level in the wild-type trophoblasts in which only DMSO was added (Fig 2C, bottom). Interestingly, similar levels of cell proliferation were observed in these two cell populations with equivalent levels of AKT Ser473 phosphorylation. These results further indicated that the upregulation of AKT activation was the possible cause of enhanced proliferation observed in *Nrk*-null trophoblasts.

### AKT was highly activated in enlarged placentas derived from *Nrk*-null mice

**Fig 3:**
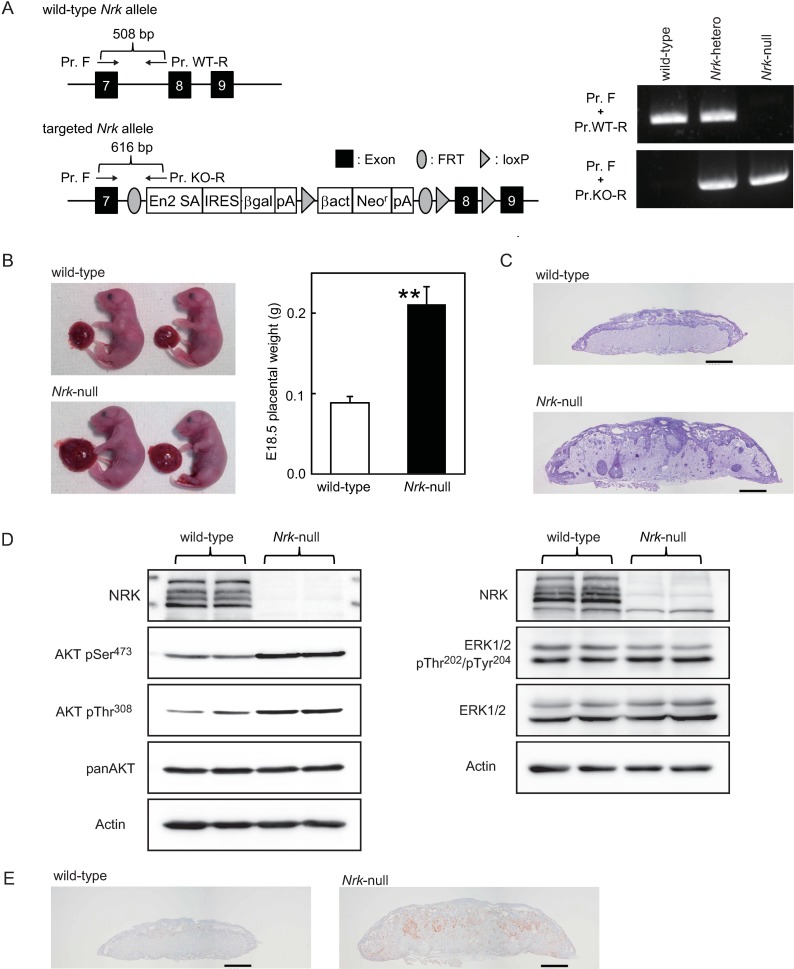
Upregulation of AKT phosphorylation in enlarged placentas derived from *Nrk*-null E18.5 mouse embryos. (A) The wild-type *Nrk* allele and the targeted *Nrk* allele are shown (left). The arrows indicate PCR primers for detection of each allele. PCR genotyping of mice (right). (B) Wild-type and *Nrk*-null mouse placentas and foetuses at E18.5 are shown (left). The placental weight was measured at E18.5 in wild-type (n = 24) and *Nrk*-null (n = 25) embryos (right). Data are presented as mean plus SD. **P<0.01 compared to wild-type. (C) PAS staining of E18.5 placental sections. Scale bars indicate 1 mm. (D) The lysates were prepared from wild-type (n = 2) and *Nrk*-null (n = 2) placentas at E18.5 and were subjected to western blot analysis. Expressions of NRK, phosphorylated AKT at Ser473, phosphorylated AKT at Thr308 and total AKT were compared between wild-type and *Nrk*-null placentas (left). Expressions of NRK, phosphorylated ERK1/2 and total ERK1/2 were compared between wild-type and *Nrk*-null placentas (right). As an internal control, β-Actin was also detected. (E) Immunostaining of E18.5 placental sections with anti-AKT pSer473 antibodies. Scale bars indicate 1 mm. Shown are cropped gels/blots. The gels/blots with indicated cropping lines are shown in S4 Fig.

To study whether *Nrk* deficiency led to aberrant activation of AKT in placentas, we developed *Nrk*-null mice from *Nrk*-null EGR-G101 ESCs. Genotypes of mice were confirmed using PCR analysis (Fig 3A). As shown in Fig 3B, placentas from *Nrk*-null embryos were significantly larger than those from wild-type controls, which was consistent with the previous observation [12]. In addition, histological analysis by PAS staining indicated the increase of glycogen positive trophoblast cells labelled by PAS in the junctional zone of *Nrk*-null placenta and the abnormal invasion of these PAS-positive cells into the labyrinth layer. Thus, NRK deficiency resulted not only in hypertrophy but also in the disorder of internal structure of placentas (Fig 3C). A western blot analysis revealed that NRK proteins were absent in *Nrk*-null placentas that were isolated from two individual embryos at E18.5 (Fig 3D). Furthermore, phosphorylation of AKT at both Ser473 and Thr308 were clearly upregulated in *Nrk*-null placentas, while total levels of AKT remained constant. In contrast, the levels of phosphorylated and total ERK1/2 proteins were similar in both wild-type and *Nrk*-null placentas. To confirm whether the cells in placenta express phosphorylated AKT at Ser473, E18.5 placental sections were stained with anti-AKT Ser473 antibodies (Fig 3E). Though wild-type placenta showed few expressions of phosphorylated AKT at Ser473, strong expressions of phosphorylated AKT at Ser473 were detected in a part of junctional zone of *Nrk*-null placenta. Thus, these results indicated that enhanced AKT phosphorylation was associated with the placentomegaly that was observed in *Nrk*-null mice and suggested that *Nrk*-regulating AKT activation was important for normal placental development.

### Establishment efficiency of trophoblast stem cell (TSC) lines from wild-type and *Nrk*-null blastocyst was equivalent

We then tried to establish TSC lines from the blastocysts obtained from *Nrk* heterozygous mutant X¯X female mice that were mated with wild-type XY male mice. TSCs are derived from trophectoderm that is a precursor of placenta that makes the outermost layer of blastocysts. Theoretically, *Nrk* deficient X¯Y TSC lines should be established at the rate of 25%, because the expected genotypes would occur in a 1:1:1:1 ratio of X¯X: XX: X¯Y: XY. Indeed, we could establish 16 TSC lines from 36 blastocysts, in which 5 (31%), 4 (25%), 4 (25%) and 3 (19%) lines were identified as genotype X¯X, XX, X¯Y or XY, respectively. These results indicated that *Nrk* deficiency did not affect the establishment efficiency of TSC lines from mouse blastocysts.

### Disruption of *Nrk* did not show obvious influence on marker gene expressions and cell morphology in undifferentiated and differentiated TSCs

**Fig 4:**
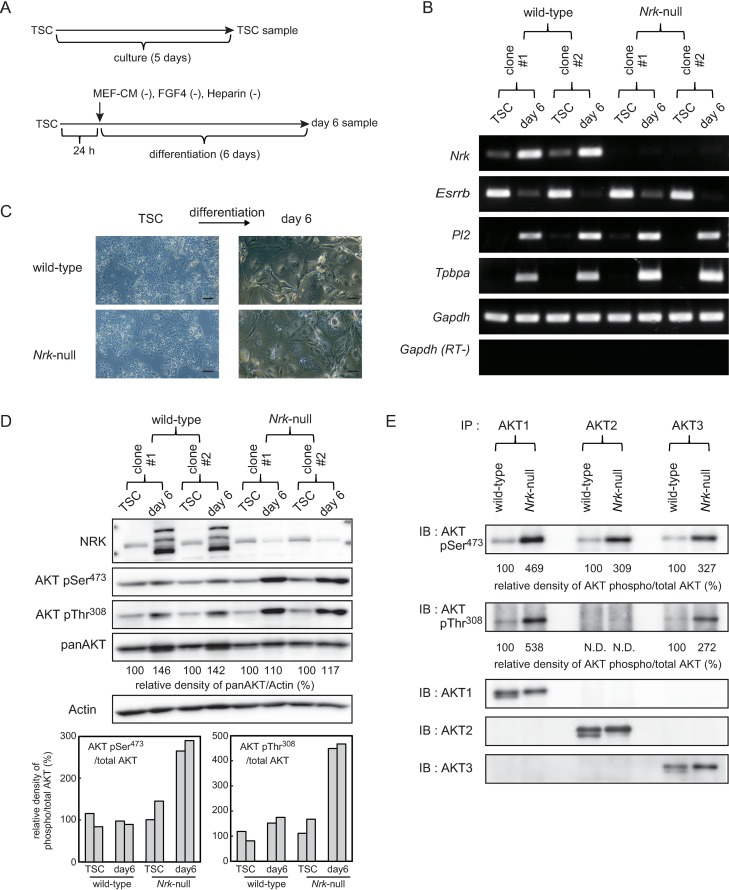
Phosphorylation of all AKT isoforms were upregulated in differentiated *Nrk*-null trophoblast stem cells. (A) The schematic representation of cell culture conditions. (B) RT-PCR analysis of undifferentiated and differentiated TSC marker gene expressions in wild-type and *Nrk*-null cells. Total RNAs were isolated from undifferentiated (TSC) and differentiated (day 6) cells and were subjected to RT-PCR amplification. As an internal control, *Gapdh* was also amplified. RT-PCR amplification of *Gapdh* was also performed in the absence of reverse transcriptase (RT-). (C) Phase-contrast photomicrographs of undifferentiated TSCs (left) and differentiated TSCs at day 6 (right) with or without *Nrk*. Scale bars indicate 200 μm. (D) Western blot analysis of whole-cell extracts from undifferentiated (TSC) and differentiated (day 6) cells. Expressions of NRK, phosphorylated AKT at Ser473, phosphorylated AKT at Thr308 and total AKT were compared between wild-type and *Nrk*-null cells. As an internal control, β-Actin was also detected (top). The relative density of total AKT vs β-Actin was analyzed and was indicated in the blot (top). The value of day 6 was normalized to the value of TSC of each clone. The relative density of phosphorylated vs total AKT was analyzed and was indicated in the graphs (bottom). The relative density was calculated by dividing the percent value for each sample by the mean percent value for wild-type TS cells, which represents 100%. (E) Immunoprecipitation was performed using anti-AKT1, anti-AKT2 and anti-AKT3 antibodies in whole-cell extracts from differentiated (day 6) TSCs. Following immunoprecipitation, immunoblot was performed using anti-AKTpSer473, anti-AKTpThr308, anti-AKT1, anti-AKT2 and anti-AKT3 antibodies. The relative density of phosphorylated vs total AKT was analyzed and was normalized to the value of wild-type. Shown are cropped gels/blots. The gels/blots with indicated cropping lines are shown in S5 and S6 Figs. TSC: trophoblast stem cell, IP: immunoprecipitation, IB: immunoblot, N.D.: not detectable.

TSCs can maintain their undifferentiated status under defined culture conditions, and can differentiate into multiple trophoblast lineages after withdrawal of FGF4, heparin and MEF-CM for 6 days. To explore the influence of *Nrk* deficiency on TSCs, samples of undifferentiated TSCs and differentiated TSCs (day 6) were prepared as described in Fig 4A. We first examined mRNA expressions of marker genes in undifferentiated TSCs and differentiated TSCs (day 6) using RT-PCR analysis (Fig 4B). We analyzed two independent clones of both wild-type and *Nrk*-null cells and confirmed that there was no big difference in the results between two cell lines with the same genotype. In wild-type cells, *Nrk* mRNA expression was faintly detected in undifferentiated TSC, and was clearly induced after 6 days of culture for differentiation, whereas *Nrk* mRNA expression was not detected in *Nrk*-null cells. TSC marker gene *Essrb* expression was highly detected in undifferentiated TSC and was decreased in differentiated TSCs (day 6) regardless of the presence or absence of *Nrk*. Both trophoblast giant cell marker gene *Pl2* and spongiotrophoblast cell marker gene *Tpbpa* were not detected in undifferentiated TSCs, and were induced after 6 days of culture for differentiation regardless of the presence or absence of *Nrk*. Moreover, cell morphology of undifferentiated TSCs and differentiated TSCs (day 6) were not affected by *Nrk* deficiency (Fig 4C). These results suggested that *Nrk* did not play a critical role in both the maintenance of undifferentiated TSCs and the differentiation process of TSCs.

### AKT phosphorylation was upregulated in differentiated TSCs (day 6) derived from *Nrk*-null TSCs

To confirm whether *Nrk* deficiency resulted in activation of AKT in differentiated TSCs (day 6) as well as in differentiated trophoblasts from ESCs (as shown in Fig 2A), western blot analysis was performed (Fig 4D top). Our results showed that NRK protein expression was clearly detected in differentiated wild-type TSCs (day 6), but not in any other cells tested, which was consistent with the mRNA expression patterns shown in Fig 4B. Furthermore, it was revealed that in addition to AKT Ser473 phosphorylation, phosphorylation of AKT Thr308 was also upregulated in differentiated *Nrk*-null TSCs (day 6). To evaluate the phosphorylation level of each site, we calculated the relative densities of phosphorylated vs total AKT and confirmed 2.8- and 4.6-fold increase of AKT phosphorylation at Ser473 and Thr308, respectively, in *Nrk*-null TSCs (Fig 4D bottom). We also noticed that the total amount of AKT was increased to 1.4-fold during differentiation in wild-type TSCs. However, the rate of AKT phosphorylation was fairly stable throughout the 6 days of differentiation in these cells. These results indicated that *Nrk* deficiency resulted in upregulation of AKT phosphorylation in newly established TSCs as well as in trophoblasts derived from ESCs.

### Phosphorylation of all AKT isoforms were upregulated in differentiated *Nrk*-null TSCs

AKT is known to be comprised of three highly homologous isoforms: AKT1, AKT2 and AKT3. Each of these isoforms presents a highly homologous structure and functional redundancy with each other [40]. Though many studies have been focused on the overall role of AKT without regard to isoform specificities, recent studies using gene-manipulated mice which have single or multiple ablation of AKT isoforms indicate the evidence of isoform-specific functions in addition to overlapping functions [41–46]. To examine which AKT isoforms were responsible for the upregulated AKT phosphorylation observed in differentiated *Nrk*-null TSCs (day 6), immunoprecipitation followed by western blot analysis was performed. As shown in Fig 4E, upregulation of serine phosphorylation was clearly observed in all three AKT isoforms derived from *Nrk*-null cells compared with those from wild-type cells. The rate of upregulation was 4.7-, 3.1- and 3.3-fold in AKT1, AKT2 and AKT3, respectively. In contrast, threonine phosphorylation was upregulated only in AKT1 (5.4-fold) and AKT3 (2.7-fold) of *Nrk*-null cells. AKT2 was not phosphorylated at the threonine residue in both wild-type and *Nrk*-null cells. Antibody-specific immunoprecipitation was properly performed, because equivalent amounts of each AKT isoform were precipitated and detected by the corresponding antibodies.

## Discussion

In this study, we found that increased phosphorylation of AKT was associated with enhanced proliferation observed in *Nrk*-null trophoblasts that were differentiated from ESCs. Furthermore, we indicated that AKT was actually hyperphosphorylated in enlarged placentas of *Nrk*-null mice. Since AKT has been shown to be important for cell growth and survival [47], it is reasonable to assume that hyperphosphorylation of AKT leads to enhanced proliferation of trophoblasts, resulting in placentomegaly. Indeed, a previous study has demonstrated the overproliferation of the spongiotrophoblast layer cells in enlarged placentas of *Nrk*-null mice. This result suggested the presence of unidentified factors that were responsible for abnormal trophoblast proliferation [12]. Moreover, it has been reported that AKT1 was widely expressed in murine placentas and that *Akt1*-deficient placentas exhibited severe hypotrophy, signifying the importance of AKT1 for normal placental development [37]. A recent study has also demonstrated that the deletion of TFAP2C resulted both in severe placental abnormalities with reduced trophoblast population and in the downregulation of AKT phosphorylation; this further indicated the pivotal role of AKT in proper trophoblast proliferation and placental development [47].

NRK induces the activation of the JNK pathway [16], promotes apoptosis [17] and phosphorylates an actin-depolymerizing protein, cofilin [18] in cultured cells that forcibly express NRK. However, these functions were not shown to be directly related to the regulation of AKT phosphorylation that we observed in this study. Moreover, it appeared that NRK did not directly phosphorylate AKT because a disruption in NRK expression resulted in hyperphosphorylation but not hypophosphorylation of AKT (Figs 2A, 3D and 4D). Because AKT phosphorylation was controlled by the balance between protein kinases and phosphatases, NRK may be involved in regulating phosphorylation by either repressing kinases or activating phosphatases in the AKT signal transduction pathway. A study demonstrated that after stimulating the PI3K pathway, AKT was rapidly and directly phosphorylated on the activation loop site residue Thr308 by PDK1 [48] and on the hydrophobic region residue Ser473 by PDK2 (mTORC2 complex) [49]. These two phosphorylation events are required for complete induction of AKT activity. Thus, repressing PDK1 or PDK2 may be a mechanism for regulating AKT activation by NRK. There have also been studies regarding molecules that negatively regulated AKT activity. Lipid phosphatases, PTEN [50] and SHIP [51], have indirectly inhibited PI3K/AKT signal transduction through the enzymatic conversion of PIP3. In contrast, other phosphatases, such as PHLPP [52] and PP2A [53], can exert direct effects by dephosphorylating AKT Thr308 and Ser473, respectively. These phosphatases could also be possible candidates that NRK affects in order to control AKT phosphorylation. In addition, a recent study demonstrated that increased production of ceramide was associated with a decline of AKT phosphorylation in trophoblasts [54]. Thus, NRK may also affect the production of ceramide or other molecules that negatively regulate AKT phosphorylation. Moreover, it is also possible that NRK could affect unknown pathways that regulate AKT phosphorylation. Because the only substrate for NRK identified to date is cofilin, and because cofilin has not been shown to be involved in regulating AKT phosphorylation, it is important to identify novel NRK substrates that affect AKT phosphorylation. Thus, further studies including the assessment of possible candidates that NRK affects in order to control AKT phosphorylation and the identification of the novel substrates for NRK will be necessary to elucidate the signal pathways underlying the regulation of AKT phosphorylation by NRK.

In addition to enhanced AKT Ser473 phosphorylation in *Nrk*-null trophoblasts, our present results revealed the induction of ERK1/2 phosphorylation in both wild-type and *Nrk*-null trophoblasts established in this study (Fig 2A). Moreover, phosphorylated ERK1/2 was also present in placentas on E18.5 (Fig 3D), suggesting the involvement of the ERK signal transduction pathway in normal placental development. Indeed, defective placental development accompanied with embryonic lethality has been reported in *Erk2*-deficient mice [38]. In contrast, *Erk1*-deficient mice have been demonstrated to be viable, fertile and of normal size [55]. Interestingly, a recent study demonstrated that the transgenic expression of *Erk1* rescues the defects in embryonic and placental development that are observed in *Erk2*-deficient mice, indicating the functional redundancy of *Erk1* and *Erk2* in mouse development [56]. By genetically modifying *Akt* and *Erk1/2* expression in the *in vitro* culture system of ESCs that were used in this study, it may be possible to further dissect the precise functions of these factors in trophoblast proliferation and differentiation.

In addition to the analysis in trophoblasts differentiated from ESCs, in this study, we examined the phosphorylation of AKT in newly established TSC lines from both wild-type and *Nrk*-null mouse blastocysts. The TSC culture system is known as an excellent tool for deciphering gene function in trophoblasts because TSCs can differentiate into trophoblast subtypes *in vitro* and contribute to trophoblast lineages *in vivo* [22]. Though ESCs and TSCs are morphologically and molecularly quite different, several studies demonstrated that ESCs can be converted to trophoblast lineages by modifying culture conditions [21] and gene expressions [19, 20]. Because no apparent difference has been shown between differentiated trophoblasts derived from TSCs and those from ESCs [19–21], it is reasonable to apply these two culture systems for better understanding the role of *Nrk* in trophoblast developments. Indeed, our present study confirmed the similar upregulation of AKT phosphorylation at Ser473 in the two culture systems. We further confirmed that all three AKT isoforms were expressed in differentiated TSCs (day 6) with almost equivalent levels between wild-type and *Nrk*-null genotypes. However, their phosphorylation levels were quite different. Although the mechanism of AKT2-speficic defect in threonine phosphorylation is not clear, we can at least say that *Nrk* deficiency strengthened complete activation of AKT1 and AKT3 and partial activation of AKT2 in differentiated TSCs (day 6). Though the precise roles of the three AKT isoforms in trophoblasts have not been elucidated, Haslinger *et al*. reported that all AKT isoforms were detected in human trophoblast cell lines [57]. They also demonstrated that gene silencing of AKT1 or AKT3 in human trophoblast cell lines resulted in the downregulation of epidermal growth factor-induced phosphorylation of total AKT at both serine and threonine residue. In contrast, gene silencing of AKT2 reduced only total AKT phosphorylation at serine residue without affecting total AKT phosphorylation at threonine residue. These results suggest that AKT2 phosphorylation at threonine residue did not occur in human trophoblasts under ordinary condition, which is consistent with our present data obtained from mouse trophoblasts. Thus, TSCs should be a useful tool to further understand the differently regulated activation mechanisms of AKT isoforms in trophoblasts.

In conclusion, this was the first study to demonstrate the involvement of *Nrk*-regulating AKT phosphorylation in trophoblast proliferation and its possible control of proper placenta formation. These findings provide important implications for our understanding of placental development. Moreover, because controlled trophoblast proliferation and differentiation are essential for placental and foetal development, our experimental systems established in this study could be useful for gaining further insights into disorders that may occur during pregnancy.

## Supporting Information

S1 Fig Gels with the cropping lines for Fig 1A, 1C and 1D. (TIF)

S2 Fig Western blot analysis of JNK. Expression of total and phosphorylated JNK showed no significant differences between wild-type and *Nrk*-null cells.
(TIF)

S3 Fig Blots with the cropping lines for Fig 2A and 2C. (TIF)

S4 Fig Gels/blots with the cropping lines for Fig 3A and 3D. (TIF)

S5 Fig Gels with the cropping lines for Fig 4B. (TIF)

S6 Fig Blots with the cropping lines for Fig 4D and 4E. (TIF)
