# Supplementary figures and images for: Nik-related kinase regulates trophoblast proliferation and placental development by modulating AKT phosphorylation
Source: PLoS One. 2017 Feb 2;12(2):e0171503. doi: 10.1371/journal.pone.0171503 (PMC5289614; doi:10.1371/journal.pone.0171503)

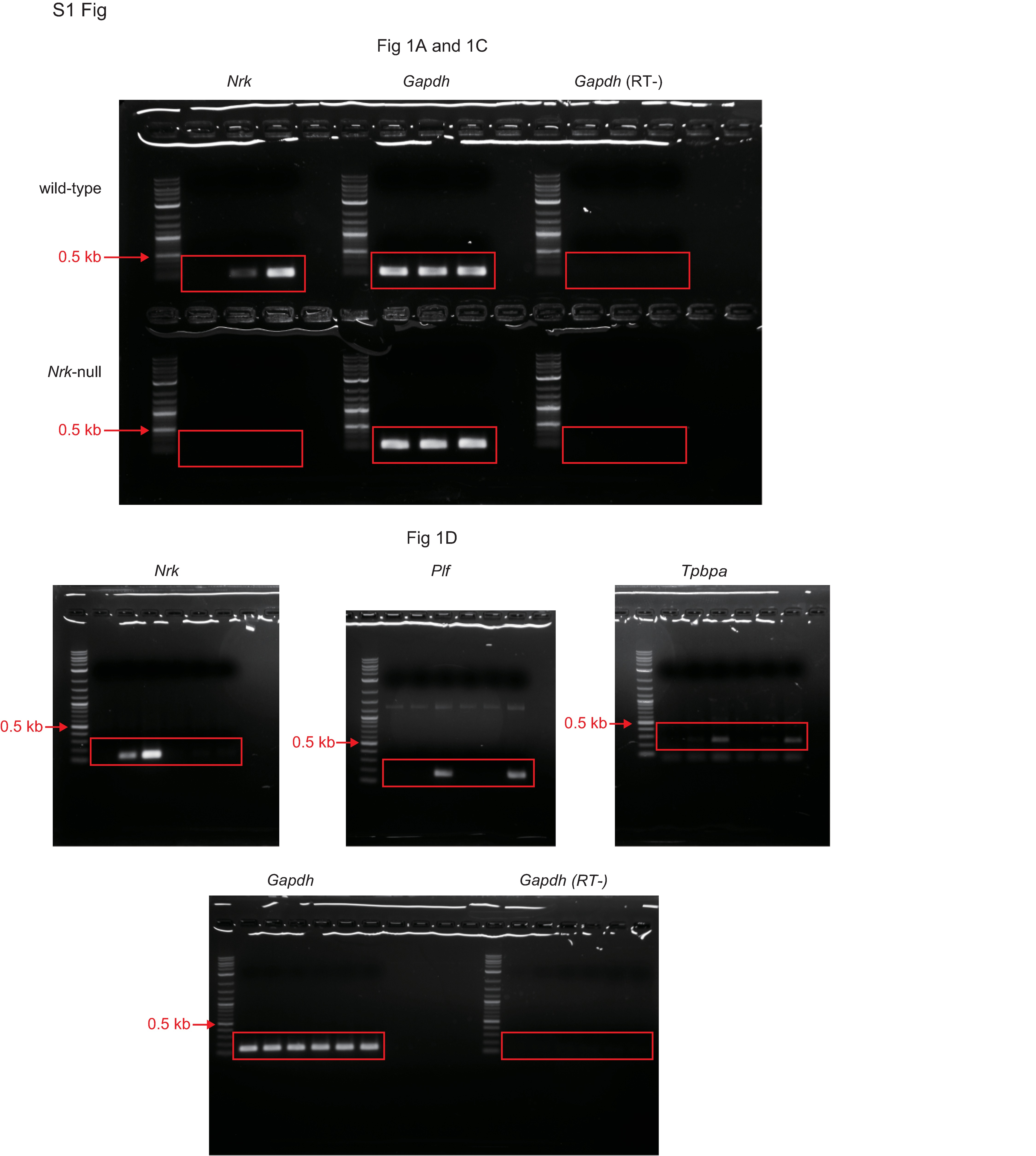

Supplement: S1 Fig — (TIF) [file pone.0171503.s001.tif]

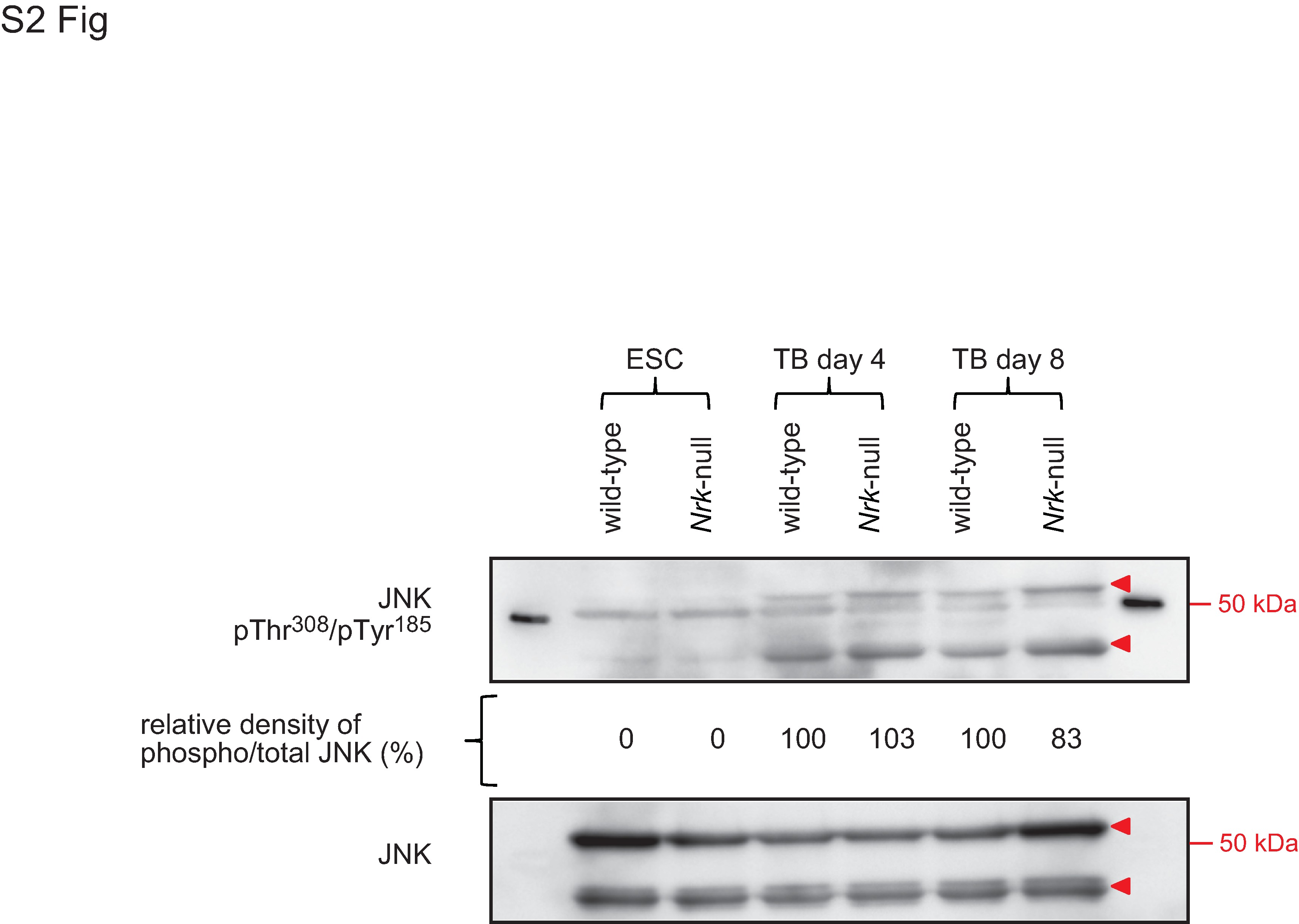

Supplement: S2 Fig — Expression of total and phosphorylated JNK showed no significant differences between wild-type and Nrk-null cells. (TIF) [file pone.0171503.s002.tif]

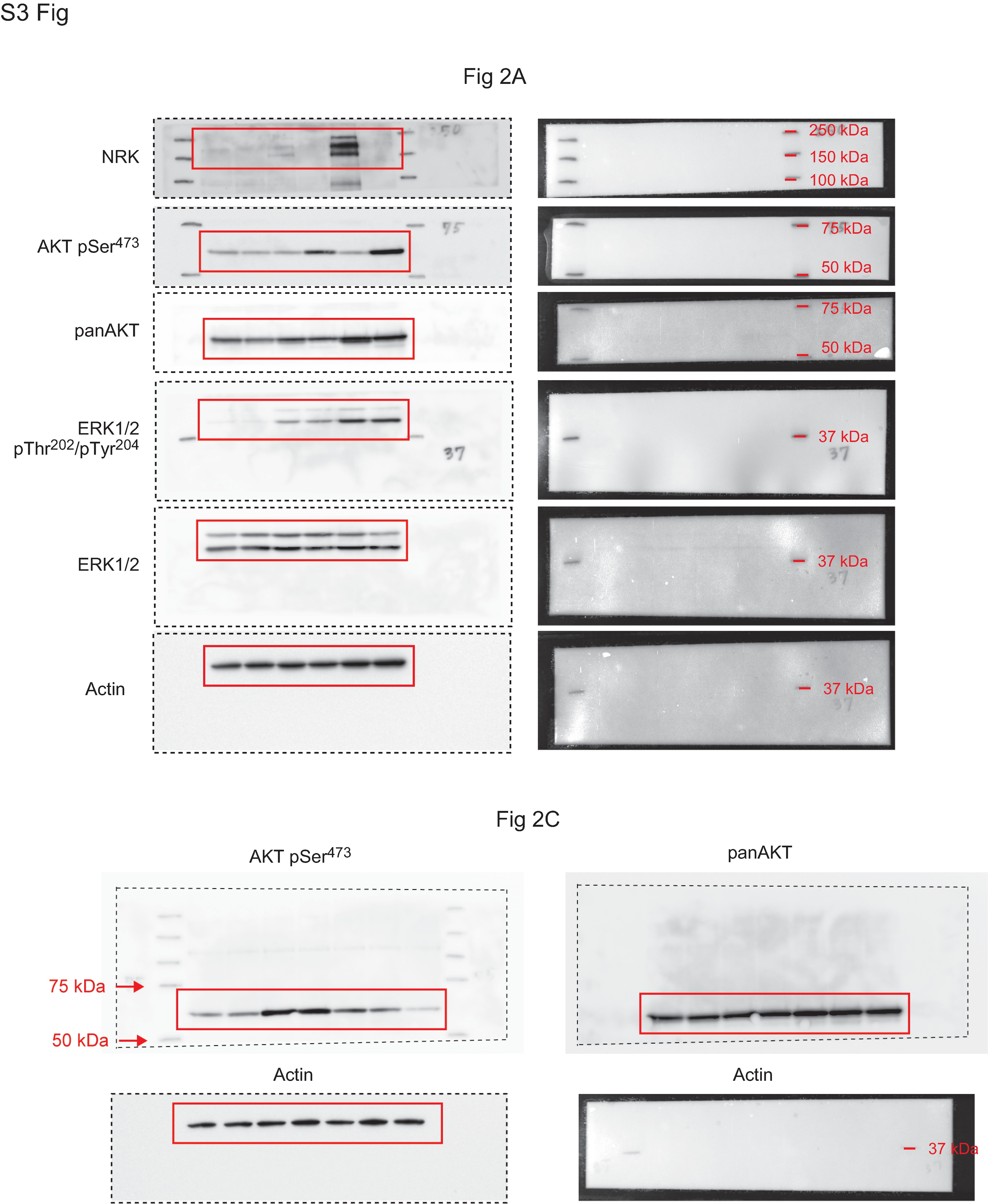

Supplement: S3 Fig — (TIF) [file pone.0171503.s003.tif]

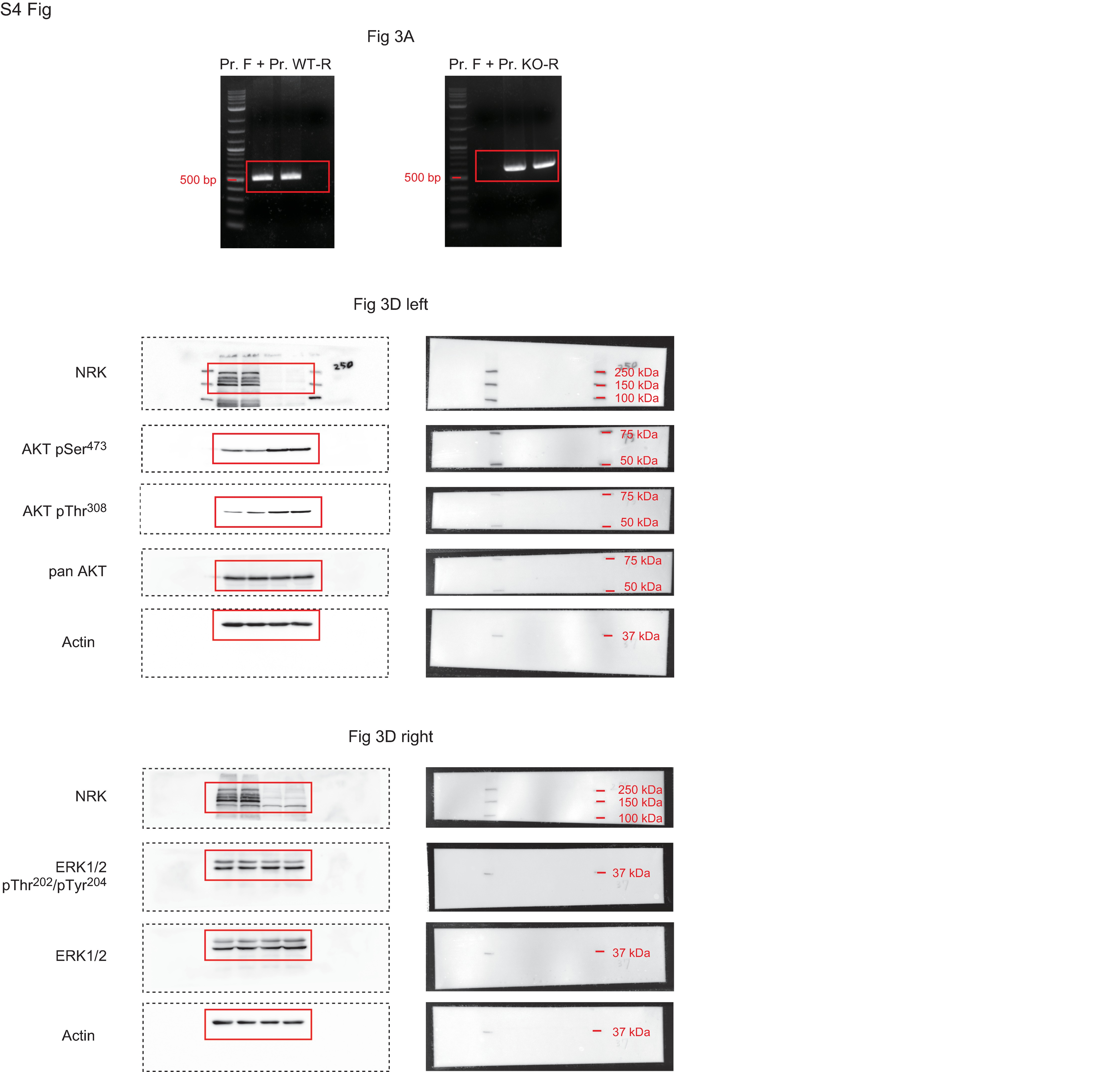

Supplement: S4 Fig — (TIF) [file pone.0171503.s004.tif]

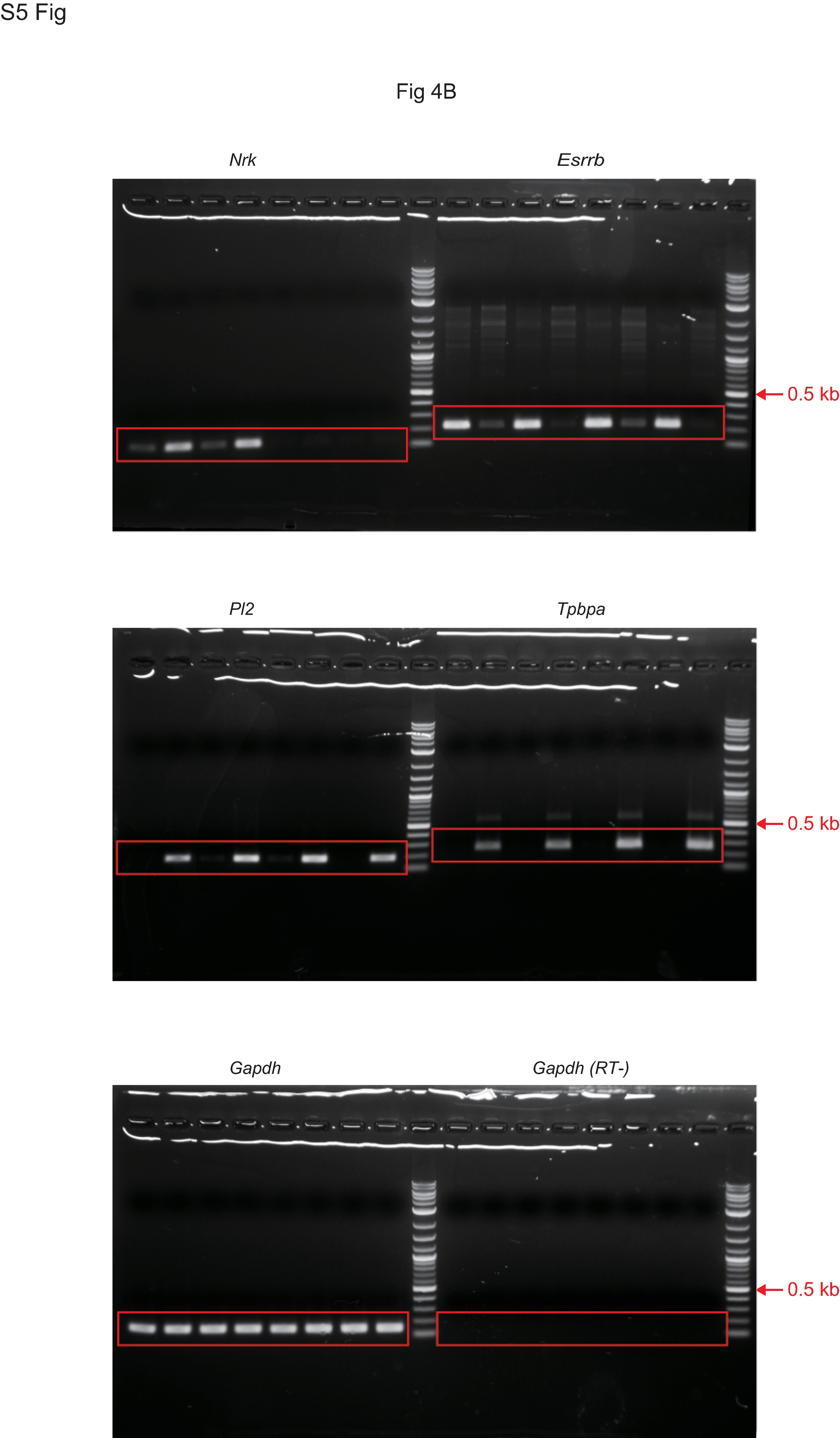

Supplement: S5 Fig — (TIF) [file pone.0171503.s005.tif]

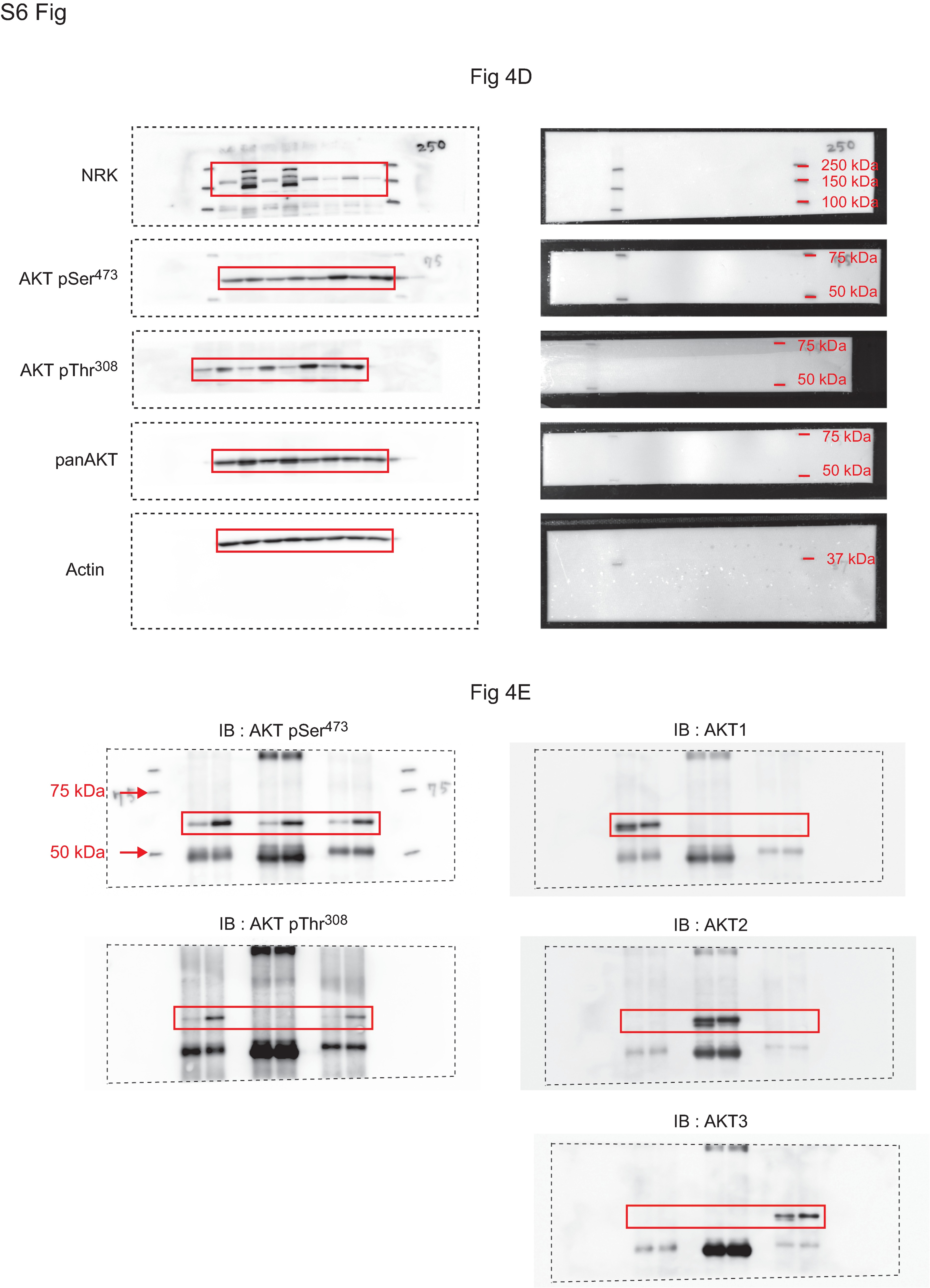

Supplement: S6 Fig — (TIF) [file pone.0171503.s006.tif]
